# Supplementary material for: Thermoelectric microscopy of magnetic skyrmions
Source: Sci Rep. 2019 Dec 5;9:18443. doi: 10.1038/s41598-019-54833-4 (PMC6895239; doi:10.1038/s41598-019-54833-4)
Supplement: Supplementary file 1 — Supplementary information [file 41598_2019_54833_MOESM1_ESM.pdf]

Supplementary Information for
Thermoelectric microscopy of magnetic skyrmions

Ryo Iguchi[1], Shinya Kasai[1,2], Kazushige Koshikawa[3], Norimichi Chinone[3], Shinsuke Suzuki[3], and Ken-ichi Uchida[1,4,5]

[1]National Institute for Materials Science, Tsukuba 305-0047, Japan.

[2]PRESTO, Japan Science and Technology Agency, Saitama 332-0012, Japan.

[3]Hamamatsu Photonics K.K., Hamamatsu 431-3196, Japan.

[4]Department of Mechanical Engineering, The University of Tokyo, Tokyo 113-8656, Japan.

[5]Center for Spintronics Research Network, Tohoku University, Sendai 980-8577, Japan.

Correspondence and requests for materials should be addressed to R.I. (e-mail: IGUCHI.Ryo@nims.go.jp)

Optical performance of focused laser beam.

Figure S1 shows the optical image of 0.5-μm-wide lines placed with 1 μm interval (0.5 μm gap). The image resolves the line and gap, indicating the resolution around 500 nm.

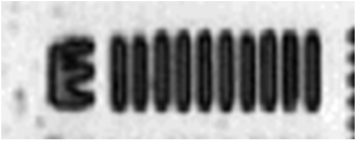

Figure S1. Optical image of 0.5-μm-wide lines with 1 μm interval.

Magneto-optical imaging of magnetic texture.

The appearance of the magnetic texture is confirmed by polar magneto-optical Kerr effect (MOKE) imaging of our sample as shown in Figure S2. A polarized white light beam was applied to the sample perpendicularly to the film plane and the reflected light distribution through an analyser is measured by an image sensor. In this configuration, the out-of-plane magnetic moment m_z is detectable. The magnetic field is applied in the out-of-plane direction. Figure S3c shows the evolution of the MOKE images with the magnetic field magnitude H_z. Similarly to the thermoelectric images shown in Fig. 3, the magnetic texture appears at $\mu_0 H_z \sim -1.6$ mT and the particle-like objects start to appear around $\mu_0 H_z \sim 3$ mT. At $\mu_0 H_z = 4.0$ mT, there can be found the isolated skyrmions, consistent with the thermoelectric imaging. By further increasing H_z, the magnetic texture disappears as the magnetisation is fully aligned along the applied field (Fig. S2b).

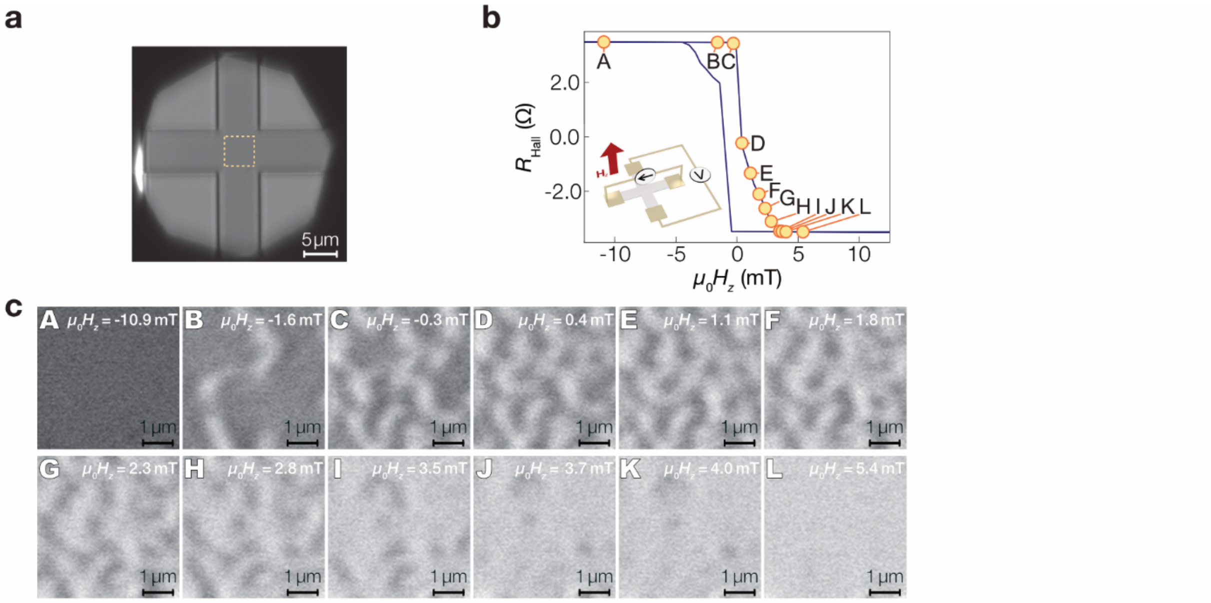

Figure S2. Magneto-optical imaging of magnetic texture. (**a**) Raw MOKE image measured at the magnetic field point A. (**b**) H_z dependence of the Hall resistance R_{Hall} of the sample system, which reflects the magnetic moment perpendicular to the plane. The MOKE images were measured at the magnetic field marked with yellow circles in the R_{Hall}-H_z curve. (**c**) MOKE images at various values of H_z. The displayed area corresponds to the yellow rectangle area in Fig. S2a. The displayed images are the differential images from a background image taken at the field point A.

Circumferential profile of thermoelectric image obtained by two-dimensional detection.

Figure S3 shows the circumferential profiles of the magnetic circular objects in the amplitude image measured at the field label H in Fig. 3b. We calculated the profiles for three objects labelled α, β, and γ (Fig. S3b). The averaged profiles are calculated using the values in the annulus with the radius of the small (large) ring being about 230 nm (360 nm). As we discussed in the Results section of the main text (Simulation of thermoelectric images due to skyrmions), the Néel skyrmions are expected to show the uniform amplitude profile along the circumference while the trivial bubbles are expected to show the nonuniform or asymmetric profile (see Fig. 4f). Figure S3c shows, however, that the profiles do not have sufficient S/N ratio for experimental distinction.

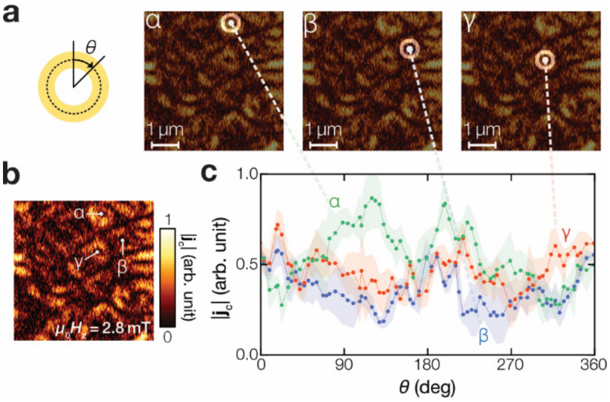

Figure S3. Circumferential profile of magnetic objects in thermoelectric image. (**a**) Schematic and regions of the annulus used for the calculation. (**b**) Amplitude image of thermoelectric microscopy (the same as Fig. 3b, H). (**c**) Amplitude profile as a function of azimuthal angle θ. The shadowed regions respectively cover the value range within the standard deviation.

Skyrmion diameter dependence of the thermoelectric images.

The diameter of the skyrmions is estimated by comparing the experimental and calculated results. Figure S4a shows the skyrmion diameter (d) dependence of the simulated thermoelectric images. By comparing the diameter of the ring-like pattern in the amplitude image at H of Fig. 3b and the calculated amplitude images, the skyrmions' diameter is found to be around 1000 nm. Interestingly, Fig. S4a indicates that although the magnitude decreases as d decreases (see Fig. S4c), the spatial variation of the $\mathbf{j}_c$ direction around the skyrmion's centre remains even when the optical laser spot size is comparable to d (Fig. S4b). This is due to the combination of steeply-changing magnetic structure of the skyrmions and spin-caloritronic phenomena. The sign of the thermoelectric signals is reversed between one and the other halves of each skymion. Thus, even if the heating diameter is comparable to the skyrmions, the shift of the heating centre from the skyrmion's centre ensures finite thermoelectric signals. We note that apart from the skyrmions' core, the spatial distribution is simply broadened following to the optical limitation.

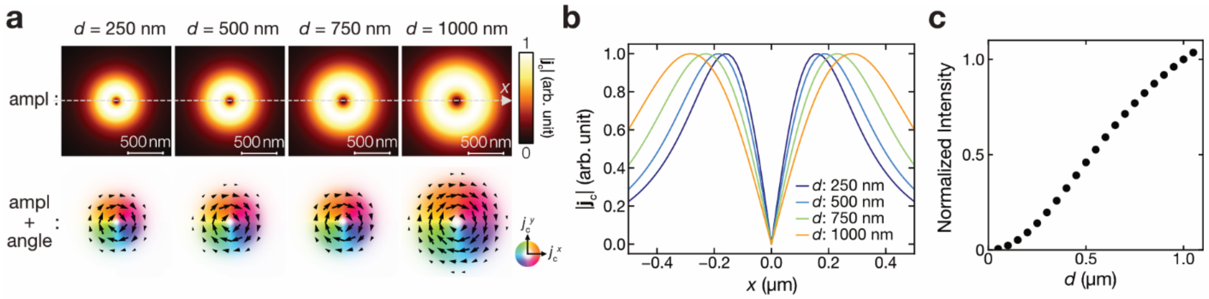

Figure S4. Temperature profiles and expected thermoelectric images. (**a**) skyrmion diameter d dependence of the thermoelectric images. The signals are normalized in each image with the maximum value, which is

plotted in Fig. S4c. (**b**) The profile of the total $|\mathbf{j}_c|$ image along the centre line for various values of d. (**c**) The maximum value of the total $|\mathbf{j}_c|$ images as a function of d, where the values are normalized at d = 1.0 μm.

Optical image of prepared sample.

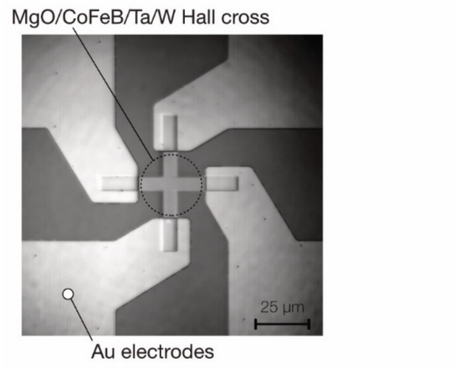

Figure S5. Optical image of the Hall cross and four Au electrodes used for two-dimensional thermoelectric current detection.

Extraction of thermoelectric image due to spin caloritronic phenomena.

The thermoelectric images shown in the main text were obtained after subtracting periodic environmental noise and background offset from the raw thermoelectric images. Firstly, the periodic noise with high spatial frequency (the wavy pattern in Fig. S6) was eliminated using a high-pass filter in the following procedure. The raw image was converted into a Fourier transformation (FT) image, the points with high wave vectors and large intensity were subtracted. Then, the FT image was converted back into the thermoelectric image. Secondly, the uniform background offset was subtracted, which is due to the electronic circuit, such as the characteristic offset of the amplifier and the thermoelectric current due to the environmental temperature difference inside the circuit. The offset value was calculated as the averaged value over the regions A-D shown in the leftmost images of Fig. S6, where A-D locate outside the Hall cross and no spin-caloritronic signals are expected. In the thermoelectric images after the offset subtraction, negative/positive signals can be found on the top/bottom of the horizontal section of the Hall cross. These signals might be due to the magnetic moments lying in-plane at the Hall cross edges and/or the asymmetric in-plane temperature gradient induced by edge illumination[1]; while at inside, the in-plane temperature gradient is radial and thus the signal is induced only when the out-of-plane magnetic moment m_z distributes asymmetrically in the heating spot, at the edges, the in-plane temperature gradient is formed only toward the other edge and thus it simply reflects $m_\perp$. In addition, similar situation can happen for edges where the electrical sensitivity changes, *i.e.* around

the boundary of the centre part of the Hall cross. We note that these parasitic signals do not affect the signals inside the cross area of the Hall cross, which we discussed in Fig. 3b.

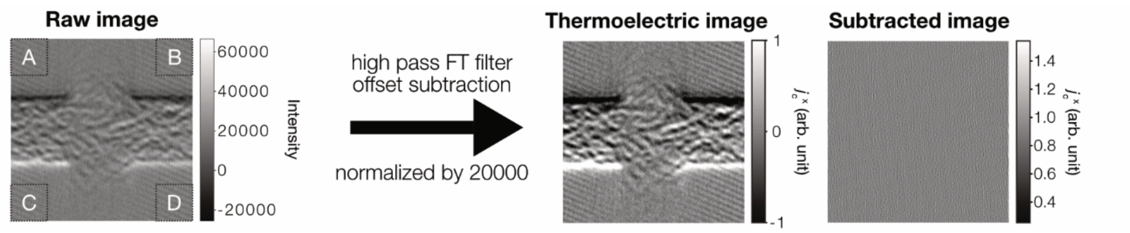

Figure S6. Schematic of signal processing. Raw, thermoelectric, and subtracted images are shown, where the subtracted image consists of the high frequency environmental noise and uniform offset. The raw image was recorded with 16-bit resolution for 32 times. The rectangles with the labels A, B, C, and D indicate the regions used for calculating the uniform offset.

Power dependence of thermoelectric image.

To check that the magnetic texture in our sample is not changed by the laser heating, we measured the laser power P_{in} dependence of the thermoelectric images. Figure S7 shows that the distribution of the thermoelectric signals does not change when $P_{\mathrm{in}} < 1.14$ mW, confirming that the experimental results shown in the main text, measured at $P_{\mathrm{in}} = 0.84$ mW, are not affected by laser light effects[2]. Note that small change in the patchy pattern can be found at 1.28 and 1.43 mW irradiation.

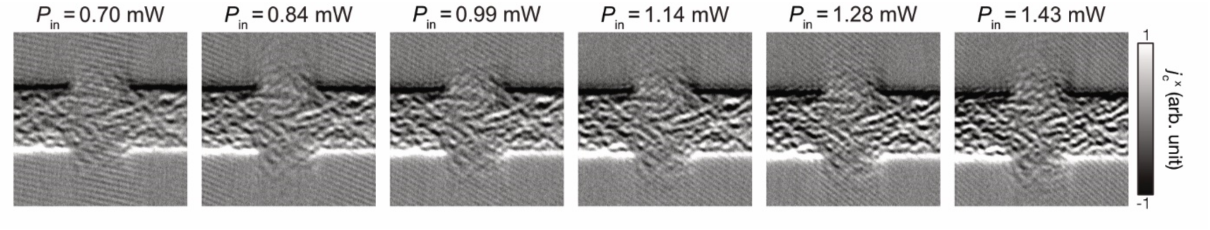

Figure S7. Irradiation power P_{in} dependence of thermoelectric images.

Estimation of the magnitude of the anomalous Nernst and spin Seebeck effects.

In order to estimate the magnitude of the ANE and SSE contributions, we compared the magnitude of the transverse thermopower in the MgO/CoFeB/Ta/W multilayer film with the in-plane (IP) and out-of-plane (OP) magnetization[3,4]. For the IP (OP) magnetized system, both the ANE and SSE appear (only the ANE appears). The sample for this measurement was made on the thermally oxidized Si substrate, where the width w is 2.0 mm and the length is 6.0 mm. The MgO/CoFeB/Ta/W film was deposited on the whole surface of

the substrate and annealed in the same manner as the device used for the thermoelectric imaging. The sample was loaded on a heat bath and cramped by a heater block, which is thermally isolated from the environment so that the entire applied heater power P_{heater} flows to the heat bath through the sample[5]. The length of the heater block l' is 5.0 mm and the thermoelectric voltage V induced within l' was measured using a voltmeter. With applying P_{heater} = 150 mW to the heater block and the magnetic field to the sample, we measured the thermoelectric voltage along the length direction. The recorded voltage V is converted into the transverse thermopower S using the relation

$$S = \frac{V}{l'\nabla T} \tag{S1}$$

by estimating the temperature gradient ∇T applied to the sample. For the IP magnetized system, ∇T is simply determined by the thermal conductivity κ of the sample layer:

$$\nabla T = \frac{P_{\text{heater}}}{\kappa l' w} \tag{S2}$$

For the OP magnetized system, ∇T is approximately determined by κ_{subs} and thickness t_{subs} of the substrate because the thermal resistance is governed by the substrate:

$$\nabla T = \frac{P_{\text{heater}}}{\kappa_{\text{subs}} l' t_{\text{subs}}} \tag{S3}$$

Figure S8 shows the magnetic field magnitude H dependence of S for the IP and OP magnetized samples. As the ANE and SSE change the sign of the output voltages when the magnetization is reversed, we extracted S_{IP} and S_{OP} based on the differences of the averaged S at the positive and negative fields, where $\mu_0|H|$ is larger than 200 mT with μ_0 being the vacuum permeability. Then, the ANE- and SSE-induced thermopowers (S_{ANE} and S_{SSE}) are calculated by the following relations

$$S_{\text{ANE}} = S_{\text{OP}} \tag{S4}$$

$$S_{\text{SSE}} = S_{\text{IP}} - S_{\text{OP}} \tag{S5}$$

The obtained values are S_{ANE} = 0.22 μV/K and S_{SSE} = –0.14 μV/K, of which the sign and magnitude are consistent with previous reports[6,7]. For the above calculation, we assumed the thermal conductivities used for the temperature profile calculation in Fig. 4a.

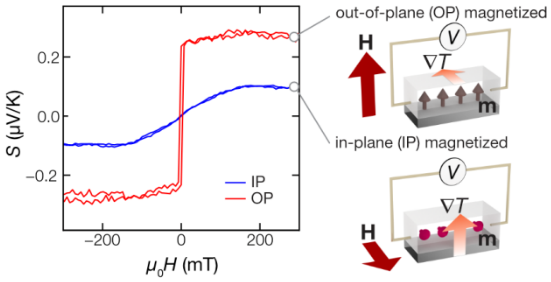

Figure S8. Magnetic field dependence of thermopower under uniform heating. The external field with the magnitude H was applied along the in-plane (IP) and out-of-plane (OP) directions of the multilayer on the rectangular-shaped substrate.

References

1. Pfitzner, E. *et al.* Near-field magneto-caloritronic nanoscopy on ferromagnetic nanostructures. *AIP Advances* **8**, 125329 (2018).
2. Koshibae, W. & Nagaosa, N. Creation of skyrmions and antiskyrmions by local heating. *Nat. Commun.* **5**, 5148 (2014).
3. Kikkawa, T. *et al.* Longitudinal spin Seebeck effect free from the proximity Nernst effect. *Phys. Rev. Lett.* **110**, 067207 (2013).
4. Kikkawa, T. *et al.* Separation of longitudinal spin Seebeck effect from anomalous Nernst effect: Determination of origin of transverse thermoelectric voltage in metal/insulator junctions. *Phys. Rev. B* **88**, 214403 (2013).
5. Iguchi, R., Uchida, K., Daimon, S. & Saitoh, E. Concomitant enhancement of the longitudinal spin Seebeck effect and the thermal conductivity in a Pt/YIG/Pt system at low temperatures. *Phys. Rev. B* **95**, 174401 (2017).
6. Lee, K.-D. *et al.* Thermoelectric signal enhancement by reconciling the spin Seebeck and anomalous Nernst effects in ferromagnet/non-magnet multilayers. *Sci. Rep.* **5**, 10249 (2015).
7. Hasegawa, K. *et al.* Material dependence of anomalous Nernst effect in perpendicularly magnetized ordered-alloy thin films. *Appl. Phys. Lett.* **106**, 252405 (2015).
